# Thumb-Sucking Habits and Oral Health: An Analysis of YouTube Content

**DOI:** 10.3390/children9020225

**Published:** 2022-02-08

**Authors:** Zaki Hakami, Prabhadevi C. Maganur, Sanjeev B. Khanagar, Sachin Naik, Khalid Alhakami, Omar A. Bawazeer, Ahmed Mohammed Alassiry, Satish Vishwanathaiah

**Affiliations:** 1 Division of Orthodontics, Department of Preventive Dental Sciences, College of Dentistry, Jazan University, Jazan 45142, Saudi Arabia; 2 Division of Pediatric Dentistry, Department of Preventive Dental Sciences, College of Dentistry, Jazan University, Jazan 45142, Saudi Arabia; prabhadevi.maganur@gmail.com; 3 Preventive Dental Science Department, College of Dentistry, King Saud Bin Abdulaziz University for Health Sciences, Riyadh 11481, Saudi Arabia; sanjeev.khanagar76@gmail.com; 4 King Abdullah International Medical Research Center, Riyadh 11481, Saudi Arabia; 5 Dental Biomaterials Research Chair, Dental Health Department, College of Applied Medical Sciences, King Saud University, Riyadh 11451, Saudi Arabia; snaik@ksu.edu.sa; 6 Department of Dental Services, King Abdulaziz Medical City, Ministry of National Guard Health Affairs, Jeddah 21423, Saudi Arabia; kalhakamik@gmail.com; 7 Orthodontic Unit, Dental Department, John Hopkins Aramco Healthcare, Dhahran 31311, Saudi Arabia; obawazeer2@gmail.com; 8 Department of Preventive Dental Sciences, Faculty of Dentistry, Najran University, Najran 55461, Saudi Arabia; amyahia@nu.edu.sa

**Keywords:** digit sucking, oral habits, social media, thumb sucking, YouTube

## Abstract

This study aims to investigate the content and usefulness of YouTube videos on** thumb-sucking habits. Methods: YouTube was systematically searched for all relevant videos on thumb sucking using primary keywords, such as thumb, finger, and digit sucking. Video information was assessed, such as the type of video, number of likes or dislikes, number of views, and duration of upload. The usefulness of videos was analyzed, and information about treatment modalities was evaluated. Results: A total of 331 YouTube videos (314 educational offerings and 17 testimonials) were included in the analysis. Individual users uploaded (36.6%), followed by healthcare professionals (30.5%). Only 4.83% of the videos were classified as having “very useful” general information content, whereas 51.1% were rated as “slightly useful.” There was no significant correlation between the usefulness score and the interaction rate, video length, or viewing rate. The videos advised a psychosocial approach and mechanical or reminder therapy in 32.33% and 25.07% of videos, respectively. Preventive methods accounted for 7.26%, and chemical treatments were discussed in 5.44% of the videos. Conclusion: Information on YouTube about thumb-sucking habits was unsatisfactory and should be improved by oral healthcare professionals and organizations.

## 1. Introduction

The stomatognathic system plays an essential role in developing head and neck structures. Oral habits are neuromuscular in action and are directly related to the stomatognathic system [1]. Habits picked up by children can cause temporary or even permanent harm to orofacial structures. The most prevalent oral habits that could cause damage include mouth breathing, digit sucking, bruxism, tongue thrusting, nail-biting, and lip biting [2,3]. The persistence of oral practices, such as thumb or tongue thrusting, until a child is three years old is considered normal. 

However, continuing these habits during later stages of life causes unwanted dental and skeletal changes that may lead to malocclusion [3,4]. Several treatment modalities can prevent or interrupt improper habits related to the oral cavity. Such interventions can even rectify the damage to orofacial structures [5]. The most useful management methods include myofunctional devices and fixed or removable appliances to stimulate and guide a new neuromuscular pattern [6]. Habits are formed from birth, and parents play a pivotal role in influencing their formation, particularly in providing health-related information and reinforcing good oral hygiene habits. Therefore, the parents’ level of knowledge can positively impact the oral health of children [7]. 

Social networking videos containing clinical and dental information are becoming increasingly popular. Of these, YouTube is one of the most prevalent digital sources of video information [2,8]. It can help people access health-related information and influence the viewers’ decision-making regarding diagnostic and treatment procedures [9,10]. Despite the usefulness of social media, incomplete or low-quality information available on these platforms can cause a patient bias toward specific treatment modalities. Thus, healthcare professionals have raised many concerns regarding the content and quality of YouTube videos [11].

To our knowledge, no studies have evaluated the content of YouTube videos on thumb-sucking habits. Hence, this study aims to review the content of videos related to thumb-sucking habits available on YouTube. The objectives are to evaluate the usefulness of these videos to inform people about thumb sucking and to evaluate the treatment modalities promoted.

## 2. Methods

### 2.1. Searching for Videos

Google Trends is the most commonly used search application in many parts of the world. This study’s search strategy included the widely used search terms for videos on YouTube, such as “thumb sucking,” “finger sucking,” and “digit sucking.” A combination of the words using the Boolean operators, such as “and” were also used. 

**Figure 1:**
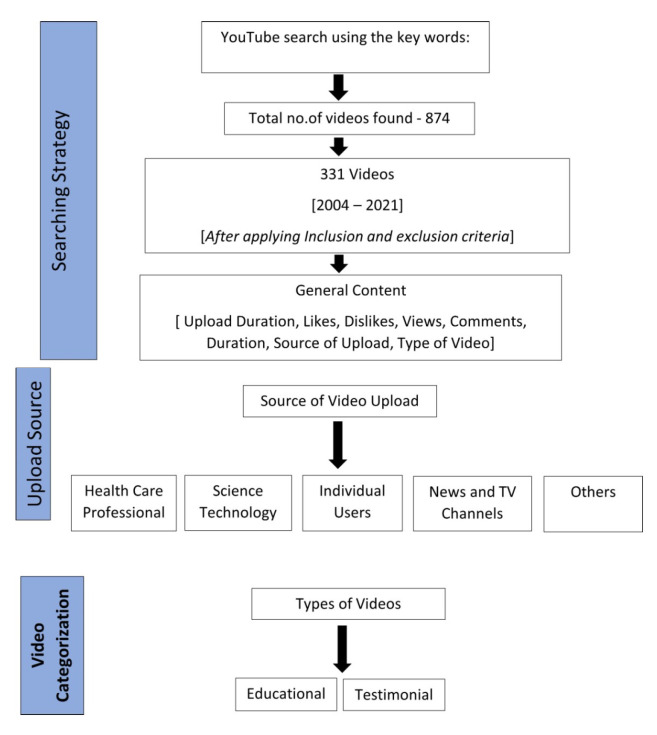
Flowchart showing the methods employed in the study.

All videos on the YouTube platform (https://www.youtube.com) accessed on 19 November 2021 were searched using the term “thumb-sucking” to evaluate any relevant information on adverse oral habits (Figure 1). After applying the inclusion and exclusion criteria, a playlist of the videos and their source locators were identified and saved. This study did not require the approval of the local ethics committee as the survey comprises data available on public platforms. 

Keywords were used to search the data, and the videos were further sorted. Videos in the English language with acceptable sound and image quality matching the keywords were included in this study. There were no restrictions on the length of the video. Those in other languages, those with poor sound quality, and duplicate videos were excluded.

### 2.2. Analysis of the Videos

All selected videos were analyzed by P.C.M, who specializes in pediatric dentistry. The extracted content was evaluated according to the duration of upload, length of the video, type of video, number of likes or dislikes, number of views, comments of viewers, and subscriptions. Depending on the person who had uploaded the video, the upload source was categorized as a healthcare professional, science and technology, individual users, news and television (TV) channels, and others. The video type was divided into educational and testimonial. Interaction with viewers was evaluated using the following interaction index and viewing rate formulas [12]:

$$\mathrm{Viewers}\text{′}\;\mathrm{interaction}\;\mathrm{index}=\frac{\mathrm{Number}\;\mathrm{of}\;\mathrm{likes}\;-\;\mathrm{Number}\;\mathrm{of}\;\mathrm{dislikes}\;}{\mathrm{Number}\;\mathrm{of}\;\mathrm{views}}\times 100$$

$$\mathrm{Viewing}\;\mathrm{rate}=\frac{\mathrm{Number}\;\mathrm{of}\;\mathrm{views}\;}{\mathrm{Number}\;\mathrm{of}\;\mathrm{days}\;\sin\mathrm{ce}\;\mathrm{upload}}\times 100$$

The videos were assessed under the following five exclusive domains: (i) etiological and risk factors, (ii) clinical features/findings, (iii) various treatment modalities and options, (iv) promoting prevention, and (v) used images of habits. Based on these domains, a usefulness score was devised (Table 1). The scores for each criterion were added together to provide an overall score, ranging from 0 to 10. Videos that did not provide any scientific information were given a score of zero and considered “not useful.” Scores of 1–3 were considered “slightly useful,” scores of 4–5 “moderately useful,” and scores of 6–10 “very useful.”

Any doubts in scoring or categorizing a video were resolved by discussing and reviewing the footage with S.V., who specializes in pediatric dentistry, and Z.H., who specializes in orthodontics until an agreed decision was reached.

### 2.3. Statistical Analysis

The data were coded and entered into a Microsoft Excel spreadsheet. Data analysis was performed using SPSS for Windows (v.20; IBM SPSS Statistics Inc., Chicago, IL, USA). Descriptive statistics included the computation of percentages, means, and standard deviations. An unpaired *t*-test was performed for quantitative data to compare two independent groups, and analysis of variance (ANOVA) was used to compare all clinical indicators within three groups quantitatively. Chi-squared and Fisher’s exact tests were applied to compare the groups. A *p*-value of less than 0.05 was considered statistically significant.

## 3. Results

The search for the term thumb-sucking” generated 874 videos in total. Among these, 331 videos were included in the final assessment after weighing the various inclusion and exclusion criteria. The included videos were published between 2004 and 2021. Each video was given a designated score with the help of the usefulness scoring system and categorized as either educational or testimonial. The upload source of each video was also noted. The total number of likes and dislikes, views, and days since upload were recorded to calculate the viewers’ interaction index and viewing rate.

Table 2 presents the distribution of usefulness categories based on video type. Most of the videos assessed were educational (94.9%), and the distribution of these videos among the usefulness categories was not significant (*p* = 0.68). A comparison between the educational and testimonial YouTube videos is provided in Table 3. The educational videos had a usefulness score of 2.71 ± 2.26, whereas the testimonial videos showed a score of 2.23 ± 2.13, with no significant difference (*p* = 0.39). There were no significant differences in the duration (*p* = 0.30), interactive index score (*p* = 0.27), and viewing rate (*p* = 0.85) between the educational and testimonial videos (Table 3).

A summary of the descriptive statistics of the video upload sources according to the usefulness categories is presented in Table 4. A comparison between the upload source (healthcare professional, individual users, news and TV channels, science and technology, and others) and the usefulness score/criteria was statistically significant (*p* = 0.001). Table 5 compares the YouTube videos based on their usefulness scores. An ANOVA was performed to analyze the correlation between the usefulness score and the interaction rate, video length, and viewing rate. These results were not statistically significant.

Different treatment modalities for thumb-sucking habits were mentioned in 70.90% of the YouTube videos (Table 6). Preventive treatment, such as the use of pacifiers, books, or distraction, was discussed in 7.26% of the videos. The most promoted treatment modality (32.33%) involved a psychological approach, comprising no scolding, an elbow guard, a thumb guard, a hand aid, a pair of gloves, a full-sleeve dress, reward therapy, and positive reinforcement. Mechanical or reminder therapy, including intraoral appliances, myofunctional devices, or orthodontics, was discussed in 25.07% of the videos, whereas chemical treatment with medications was the least recommended (5.44%).

## 4. Discussion

YouTube contains a relatively low number of enlightening and enriching videos on thumb sucking. Most of the videos assessed focused on the etiology, diagnosis, prevention, and various treatment recommendations for these habits. Few were about the experiences of patients, which is inconsistent with previous studies that have found that most videos related to healthcare topics on YouTube were uploaded by patients [13,14].

YouTube offers free uploading of oral health-related videos that have not been peer-reviewed, and the source of information is not always clear. Therefore, viewers might be unsure of the credibility of videos [15]. There are no standard methods to analyze the quality of online videos. Accordingly, in this study, a scale of 0–10 was developed based on etiological or risk factors, clinical features and findings, various treatment modalities and options, promoted prevention, and various treatment modalities. The scores were further divided into four categories. 

Several previous studies have adopted the Global Quality Scale Field’s video information and quality index [16,17]. Few studies have mentioned the source of videos and classified YouTube videos as educational or testimonial [12]. The current study showed a significant relationship between the usefulness of videos and the upload source. We observed that many educational videos were uploaded from various sources, of which only a few were “extremely or very useful,” and most were “slightly useful.” The remaining were “moderately useful.” 

Either healthcare professionals or individual users uploaded the “very useful” ones. These educational videos on thumb sucking explained the etiological factors, recorded actual cases, and demonstrated the manifestations of open bite and thumb dermatology. Very few focused on the various treatment modalities available for thumb sucking. However, all the videos might motivate parents to seek treatment.

After receiving permission, dentists can legally upload videos of patients regarding their personal experience in managing thumb-sucking habits, difficulties faced because of the practice, and how the treatment improved their life. These videos are highly informative and can motivate other patients to come forward and seek treatment [9,18]. 

Simsek et al. [2] reported that the videos on oral habits have been uploaded mostly by dentists for information and were generally moderate or good audio-visual quality. However, in our study, the videos uploaded by healthcare professionals did not provide adequate information according to the usefulness scoring system. Users uploaded few testimonial videos that included the patient’s point of view. The majority were tribute recordings discussing the various hardships individuals face while seeking help. Out of 16 videos, only one was considered “extremely useful” in the study. These results suggest that very few people have expressed their views on thumb sucking or elaborated upon their satisfaction and success rates with the various treatment modalities available.

YouTube videos are ranked high or low based on the number of views and likes or dislikes received. Content-rich videos could garner a low number of likes, whereas something sensational (although not factual) could accumulate a high number of likes. This makes the search process considerably more challenging, as many useful videos are less popular [19]. Pearson’s correlation analysis showed no significant relationship between the interaction index, duration of videos, and usefulness of videos. Similar results have been observed in various medical and dental discussions [12,17,19]. 

Studies have suggested that uploaded videos should run for a short duration for better results and understanding [20]. In this study, the viewing rate of educational videos was higher than that of testimonial videos; this might be because of the low number of testimonial videos uploaded by users. Previous studies have shown that testimonial videos interest viewers, as the content in these videos is more helpful in answering the majority of their questions [21]. However, most of the videos did not prove very useful from our analysis. Simsek et al. reported that the top-ranked videos had a low information content and noticed that the relevance did not reflect the actual content [2]. 

## 5. Limitations

YouTube is a highly dynamic search engine, and results continuously change over time because of viewers’ interests and video-watching habits. Variables, such as the viewing rate and number of likes or dislikes, can vary, as can the most used keywords for “thumb-sucking” sourced by Google Trends. Such changes could result in different search results and data.

## 6. Conclusions

YouTube contains a plethora of resources and information on thumb sucking. We found that very few videos explained all relevant details on thumb-sucking practices that would be useful for viewers. Most of the videos were educational and discussed the etiological features and clinical findings. Very few explained how to improve the management of the habit. There were far fewer testimonial videos than there were educational ones. 

Only four videos were found that provided information on the patient–clinician relationship and patient experiences regarding their treatment. The patient–clinician relationship and effectiveness of treatment were explained in most testimonial videos. Most of the useful videos were uploaded by healthcare professionals and individual users. Healthcare professionals and individuals should be encouraged to upload educational and testimonial videos. These will help parents and patients to develop a better understanding of thumb-sucking habits.

## Figures and Tables

**Table 1:** Usefulness scoring system.

No.	Criteria	Score
1	Video mentioned what thumb sucking is	1
2	Video mentioned an etiological factor	1
3	Video mentioned extraoral features and effects on the maxilla	1
4	Video mentioned extraoral features and effects on the mandible	1
5	Video used images related to the habits	1
6	Promoted prevention/early diagnosis	1
7	Video mentioned removable and fixed appliance therapy	1
8	Video mentioned reminder therapy/myofunctional therapy	1
9	Video mentioned correction by orthodontic treatment	1
10	Video mentioned various other treatment modalities	1

**Table 2:** Distribution of the usefulness scoring system of YouTube videos according to video type.

Usefulness Score	Video Type		Total *N* (%)
	Educational *N* (%)	Testimonial *N* (%)	
Not useful	63 (94.02%)	4 (5.97%)	67 (20.24%)
Slightly useful	159 (94.08%)	10 (5.91%)	169 (51.05%)
Moderately useful	77 (96.25%)	2 (2.5%)	80 (24.16%)
Very useful	15 (93.75%)	1 (6.25%)	16 (4.83%)
Total	314 (94.9%)	17 (5.1%)	331 (100%)

Chi-squared test *p* =0.68; *N* = number of videos.

**Table 3:** Comparison between educational and testimonial YouTube videos about thumb-sucking habits.

Characteristics	Video Type (Mean ± SD)		
	Educational	Testimonial	*p*-Value
Interaction index score	0.91 ± 1.81	0.42 ± 0.98	0.27
Video duration (minutes)	3.99 ± 6.47	2.37 ± 1.56	0.30
Viewing rate	21,093.63 ± 120,970.87	26,455.64 ± 39,935.50	0.85
Usefulness score	2.71 ± 2.26	2.23 ± 2.13	0.39

Unpaired t-test; SD = standard deviation.

**Table 4:** Distribution of the upload source of YouTube videos according to the usefulness categories.

Upload Source	Usefulness Score *N* (%)				Total *N* (%)
	Very Useful	Moderately Useful	Slightly Useful	Not Useful	
Healthcare professionals	11 (10.9%)	36 (35.6%)	48 (47.5%)	6 (5.9%)	101 (30.5%)
Individual users	4 (3.3%)	16 (13.2%)	67 (55.4%)	34 (28.1%)	121 (36.6%)
News and TV channels	1 (1.5%)	14 (21.5%)	29 (44.6%)	21 (32.3%)	65 (19.6%)
Science and technology	0 (0.0%)	8 (36.4%)	14 (63.6%)	0 (0.0%)	22 (6.6%)
Others	0 (0.0%)	6 (27.3%)	11 (50%)	5 (22.7%)	22 (6.6%)
Total	16 (34.8%)	80 (24.2%)	169 (51.1%)	66 (19.9%)	331 (100%)

Fisher’s exact test *p* = 0.001; *N* = number of videos.

**Table 5:** Comparison of the usefulness categories of YouTube videos (mean ± SD).

	Very Useful	Moderately Useful	Slightly Useful	Not Useful	*p*-Value
Interaction rate	1.64 ± 2.15	0.86 ± 1.39	0.87 ± 2.08	0.78 ± 1.19	0.37
Video length	6.29 ± 5.13	3.35 ± 3.97	4.3 ± 7.49	2.99 ± 5.47	0.17
Viewing rate	21,125.1±62,712.61	13,669.22 ± 75,079.65	8327.709 ± 43,980.04	690.68 ± 1547.75	0.39

One-way analysis of variance.

**Table 6:** Treatment modalities recommended in the assessed YouTube videos.

Management	Management Type	*N* (%)	Total *N* (%)
Preventive treatment	Pacifiers	9 (2.72%)	24 (7.26%)
	Books	12 (3.63%)	
	Distraction	3 (0.90%)	
Psychological approach	No scolding	34 (10.28%)	107 (32.33%)
	Elbow guard	12 (3.63%)	
	Thumb guard	28 (8.46%)	
	Hand aid	4 (1.20%)	
	Gloves	4 (1.20%)	
	Full-sleeve dress	12 (3.63%)	
	Reward therapy/positive reinforcement	13 (3.93%)	
Chemical treatment	Medications	18 (5.44%)	18 (5.44%)
Mechanical/reminder therapy	Appliance	46 (13.9%)	83 (25.07%)
	Myofunctional	18 (5.44%)	
	Orthodontics	19 (5.74%)	

## Data Availability

The data will be provided upon reasonable request.
